# Mr. Strowger, in Answer to Harlestonensis

**Published:** 1808-08

**Authors:** S. Strowger

**Affiliations:** Harleston


### *To the Editors of the Medical and Physical Journal.*

Gentlemen,

I Observed in one of your Journals some observations upon the exhibition of Mercury in Typhus Fever, by an author who calls himself Harlestonensis. The glaring inconsistencies of which his letter is composed, that arrested my attention, I cannot pass over without briefly observing upon. The manifestations of this gentleman's too good opinion of himself, must arise from a principle incompatible with that which would have influenced him to a more just and liberal one of others. He begins, by accusing medical men in general, of advancing absurd and preposterous opinions, only because they are new and singular; and he tells us, "that we are not to set those things down for facts to be depended upon in the application of medicine, which are supported only by a case or two of uncertain benefit." It appears very extraordinary, that in this liberal survey of what he conceives to be the erroneous part of his medical brethren, he should overlook a character so conspicuous as himself; that he should commit himself in the very same error, even when he was censuring it in them, for in the following paragraph he takes upon himself to decide for us upon the important question of the propriety of mercury in typhus from a single case of uncertainty! When we see contradiction opposed to experience, by one whose professional situation must necessarily render his pretensions to medical acquirement too questionable to be admitted without investigation, we cannot feel ourselves under the influence of those unsatisfactory assertions, which, on this occasion, it was but reasonable to expect he would have given validity to by some practical observations on the general effects of mercury in typhus; but, on the contrary, the symptoms of the single case, recorded for our satisfaction, are not accounted for, nor has he informed us what in that case he attributes to the mercury! Indeed, I have been puzzled to discover this gentleman's object in writing, for were we inclined to establish our opinions from the circumstances of a single case, this is an ill chosen one to impress us with objections to the practice, as there appears nothing at all satisfactory for or against it.—Then what have we to influence us to lay it aside, or to entertain a worse opinion of it? We are simply told it is detrimental! He must be aware that naked assertion, unaccompanied companied by argument, will not avail in the world of science any more than contradiction without proof; he surely did not think of assuming to himself a consequence sufficient to abolish a plan of medicine, suggested by the highest medical authority, adopted from sound and rational theory, and established by the result of experience. Every practitioner knows that typhus may prevail in one place in a very considerable degree, and that, under almost any plan of medicine, they may generally do well. At the same time, in another part, at the distance of only a few miles, it prevails apparently under similar circumstances, (but probably under the influence of causes not understood, that varies its nature and degree, and governs its precarious termination) and upon whatever plan of medicine, however well directed, a considerable number of these may die.

On one occasion, a medicine might receive unmerited applause, and on the other, perhaps, an unworthy condemnation. How difficult then to judge from a single case. I mean by this only to intimate, that the benefits of experience are no where more essentially necessary than in the formation of a correct judgment of the importance of any medicines in typhus fever, more especially to decide upon their claims to superiority. But, under the disadvantages of a short experience, and a very confined practice, the inferences of Harlestonensis must be premature, and his decisions subject to error. In another part of this author's letter, he compares mercury in typhus to a long catalogue of disreputable medicines, which he begins with digitalis; this invaluable medicine I can tell him, was never in more general use than in the present day. I would ask if his practice have furnished him with a number of those cases in which it is particularly recommended, and whether he has employed it invariably without success? for if it has succeeded in only one of those cases, that before the introduction of this medicine, were left without a remedy, and that generally ended only in death, it would be ungrateful indeed in his unwarrantable displeasure, to snatch it from its exalted place in medical estimation, and hurl it down to swell the contemptible list of obsoletes. My principal object here, is to recommend to this gentleman, instead of Harlestonensis, hereafter to take the name of Taylor, that his future productions may not be attributed to those who do not wish to rob him of their merits.

S. STROWGER.

*Harleston, July,* 1808.

